# Experiential Diversity: New Measurement Approaches and New Insights into Daily Sleep and Cognition

**DOI:** 10.1093/geroni/igaf122.1601

**Published:** 2025-12-31

**Authors:** Soomi Lee, Rachel Koffer, Martin Sliwinski

## Abstract

Research on experiential diversity (i.e., variety) has burgeoned in recent years, yet most studies remain at the between-person analyses (e.g., comparing individuals with greater activity diversity to those with lower activity diversity). With the rise of intensive longitudinal data, researchers now have the opportunity to quantify daily experiential diversity and examine its associations with daily health outcomes. These methodological advancements coincide with theoretical advancements highlighting the importance of sociohistorical context, and invite new approaches to assessing daily experiential diversity beyond the between-person entropy and other diversity indices. This symposium brings together five presentations to understand the dynamics among daily activity diversity, nightly sleep, and ambulatory cognition, while also exploring emerging domains and innovative methods for assessing daily experiential diversity. Lee presents the associations of nightly sleep quantity and quality with day-level activity diversity, including age-related differences in these associations. Drewelies examines the associations between daily subjective working memory and within- and between-person activity diversity in older adults. Bielak uses 14-day momentary objective cognitive tests (4 times/day) and their associations with activity variety (a composite sum) at the between-person and within-person levels. Clancy focuses on a new domain of experiential diversity, diversity in emotional support, and examines its sociohistorical, and age-related trends. Koffer proposes a sequence analysis approach to daily activity diversity, showing how daily sequences are associated with entropy, age, and sleep as a daily health indicator. The Discussant, Sliwinski, will integrate these findings and discuss the implications of experiential diversity for health and well-being across the adult life span.

